# Influence of the Ultrasonic Treatment on the Properties of Polybutylene Adipate Terephthalate, Modified by Antimicrobial Additive

**DOI:** 10.3390/polym12102412

**Published:** 2020-10-19

**Authors:** Irina Kirsh, Yuliya Frolova, Olga Beznaeva, Olga Bannikova, Marina Gubanova, Isabella Tveritnikova, Valentina Romanova, Yulia Filinskaya

**Affiliations:** 1 Scientific and Educational Center Advanced Packaging Materials and Recycling Technologies, Center of the Collective Use, Moscow State University of Food Production, 125080 Moscow, Russia; bannikovaoa@mgupp.ru (O.B.); gubanovami@mgupp.ru (M.G.); iza-1995@bk.ru (I.T.); bwal1307@mgupp.ru (V.R.); 2 Laboratory of Food Biotechnology and Specialized Products, Federal Research Center of Nutrition and Biotechnology, 109240 Moscow, Russia; Y.operarius@yandex.ru; 3 Department of Automated Control Systems, Moscow State University of Technologies and Management K.G. Razumovsky, 109004 Moscow, Russia; juliyafilin@rambler.ru

**Keywords:** polybutylene adipate terephthalate, birch bark extract, antimicrobial properties, biodegradation, extrusion, ultrasonic treatment

## Abstract

Particular attention is paid to biodegradable materials from the environmental point of view and antimicrobial materials that ensure the microbiological safety of packaged products. The aim of the work was to study the properties of the composition, based on biodegradable polybutylene adipate terephthalate (PBAT) and the antimicrobial additive—birch bark extract (BBE). Test samples of materials were obtained on the laboratory extruder by extrusion with ultrasonic treatment of the melt. The concentration of the antimicrobial additive in the polymer matrix was 1 wt %. A complex research was carried out to study the structural, physico–mechanical characteristics, antimicrobial properties and biodegradability of the modified PBAT. Comparative assessment of the physico–mechanical characteristics of samples based on PBAT showed that the strength and elongation at break indices slightly decrease when the ultrasonic treatment of the melt is introduced. It was found out, that the antimicrobial additive in the composition of the polymer matrix at the concentration of 1 wt % has a static effect on the development of microorganisms on the surface of the studied modified films. Studies of the biodegradability of modified PBAT by composting for 4 months have shown that the decomposition period of modified materials increased, compared to pure PBAT. The developed modified polymer material can be recommended as an alternative replacement for materials based on polyethylene for food packaging.

## 1. Introduction

Currently, in the production of plastics, there is an obvious trend towards innovation and environmental friendliness of products [1,2]. Medicine [3,4] and packaging industry [5] are some of the numerous industries where various plastics are in demand. At the same time, the problem of environmental pollution is aggravated every year, including the consumption of these materials [6], therefore, the direction of biodegradable polymers is actively developing—polymers, capable of rapid biodegradation under the influence of environmental factors and microorganisms and having properties similar to traditional polymers [7].

**Figure 1:**
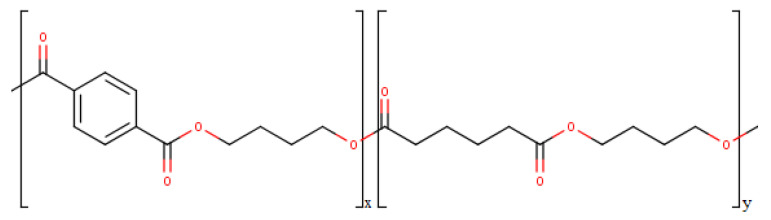
Structure of polybutylene adipate terephthalate (PBAT).

According to the report of European Bioplastics (EUBP, Germany) and nova-Institut (Germany) “Bio-based Building Blocks and Polymers—Global Capacities, Production and Trends 2019–2024”, the world market for all types of polymers in 2019 amounted to 478 million tons, while the share of bioplastics produced was approximately 1%. According to experts, the world capacity for the production of biopolymers will reach about 2.43 million tons per year by 2024, which is 15.2% more compared to 2019 [8]. One of the widely known and promising bioplastics is polybutylene adipate terephthalate (PBAT). In the segment of biodegradable materials in 2019, the share of PBAT was 13.4%. According to experts from European Bioplastics (EUBP, Germany) and nova-Institut (Germany), production capacities for the production of bioplastics increased in 2018–2019 due to the expansion of the production of PBAT [8]. PBAT refers to aliphatic-aromatic copolyesters, produced as the result of polycondensation between 1,4-butanediol, adipic acid, and terephthalic acid [9]. The structure of the PBAT block copolymer is shown in Figure 1.

The combination of starting materials used makes it possible to obtain a polymer with high flexibility, excellent impact strength and elongation at break (over 500%), good melting ability (melting point 115–125 °C) and good biodegradability [9]. The rate of biodegradation of PBAT depends on the content of terephthalic acid [9]. PBAT is subject to enzymatic degradation and is suitable for composting [9,10]. Good deformation and strength characteristics and biodegradability allow PBAT to be the alternative replacement for plastic packaging [9], in particular, polyethylene.

Packaging is an integral part of any food product, which ensures its quality and safety. The use of biodegradable packaging, alternative to traditional polymeric materials, will reduce the amount of hardly degradable polymers, which will reduce the negative impact on the environment [11]. At the same time, to ensure the quality and safety of food products, packaging must have antimicrobial properties, since the most common is microbiological contamination of food products [12]. Bacteria, fungi, parasites, and viruses can act as sources of microbial spoilage of food products [13]. In this regard, it became necessary to create packaging materials with antimicrobial properties, which remains relevant today. Today there are many developed antimicrobial polymeric materials [14], where substances of natural origin [15,16,17] and synthetic origin [18], including nanoparticles [19,20], act as antimicrobial components. Modification of PBAT will make it possible to obtain biodegradable packaging materials with antimicrobial properties [21]. One of the successful additions to PBAT was oregano essential oil, described in [22]. Packaging based on PBAT with oregano essential oil was used for packaging fish fillets, which, in terms of microbiological indicators, increased the shelf life of packaged products by 10 days. Among the variety of natural antimicrobial additives with antibacterial, antiviral, anti-inflammatory, antimutagenic properties, as well as mold resistance, it seems possible to use birch bark extract (BBE) to modify packaging materials [23,24]. In the studies researches [25,26] of antibacterial properties and resistance of birch bark extract to mold are presented. With wide range of antimicrobial activity BBE can be used to create materials with antimicrobial properties. Also, BBE is resistant to oxygen and sunlight, nontoxic [24], which allows it to be used in polymeric materials, in contact with food. In the process of introduction of modifiers into the composition of the polymer matrix difficulties, connected with melting temperature of the components used, can appear. The melting temperature of components should be close to the melting temperature of the polymer to prevent destruction of modifiers during processing. The high temperature of melting (240–260 °C) of BBE [27] allows it to be used in the extrusion process of obtaining modified polymer materials. Another technological difficulty appearing in the process of extrusion is the uneven distribution of component, which is introduced in the polymer matrix. Using of ultrasonic treatment of polymer melt allows getting more uniform distribution of modifier in the polymer matrix. Ultrasonic treatment of polymer melt is used to solve various scientific and technical problems, in particular, the use of ultrasonic treatment leads to homogenization, increases density of forming materials, reduces the surface tension at the interface between melt and matrix, which increases productivity [28]. In some researches [28,29,30,31] the effect of ultrasonic treatment on the extrusion performance of polyolefins (HDPE, LDPE, LLDPE), also polystyrene (PS) and blends HDPE/PS is presented. Regardless of the polymer matrix ultrasonic treatment reduced the viscosity of polymer melts, improved productivity and led to better compatibility of polymer blends, as well as smoother surface layer compared to non-sonicated materials. In this way, the use of ultrasonic treatment can improve the compatibility of filled polymer materials and promote more uniform distribution of additives in the polymer matrix, which provides the stability of the properties of the resulting materials. It was also found out that ultrasonic treatment of melts of polymer compositions leads to accelerated destruction of the polymer matrix due to the increase in oxygen-containing groups in the polymer matrix and the increase in water absorption [32]. Based on the above, the purpose of this study was to develop a modified biodegradable material based on PBAT with antimicrobial properties.

## 2. Materials and Methods

### 2.1. Materials

The objects of study were biopolymer polybutylene adipate terephthalate (PBAT), Easter Bio TH 801 (Eastman Chemical, Kingsport, TN, USA); birch bark extract (BBE) (Birch world, St. Petersburg, Russia), registered as a biologically active additive in the state register (certificate No. 77.99.23.3.U.3440.4.08 from 29.04.2008). As nutrient media for microbiological studies, dry nutrient medium Sabouraund (State Scientific Center for Nuclear Medicine, Obolensk, Russia), dry culture medium of Czapek-Dox (State Scientific Center for Microbiological Research, Obolensk, Russia), and meat-peptone agar (State Scientific Center for Nuclear Medicine, Obolensk, Russia) were used. All reagents, used for the analysis of material properties, were of analytical purity.

### 2.2. Polymer Materials Production Technology

Samples of polymer composite materials (PCM) based on PBAT and BBE were obtained during the work. The concentration of BBE in PCM based on PBAT was 1.0 wt %. For performance comparison, samples based on pure PBAT were prepared as control samples. The samples were obtained with and without the ultrasonic treatment of melts of polymer compositions.

At the first stage, PCM granules were obtained, at the second, film materials. For this, we used the laboratory extrusion unit with the ultrasonic treatment of the melt by extrusion, specially developed at the FSBEI VO “MGUPP”.

**Figure 2:**
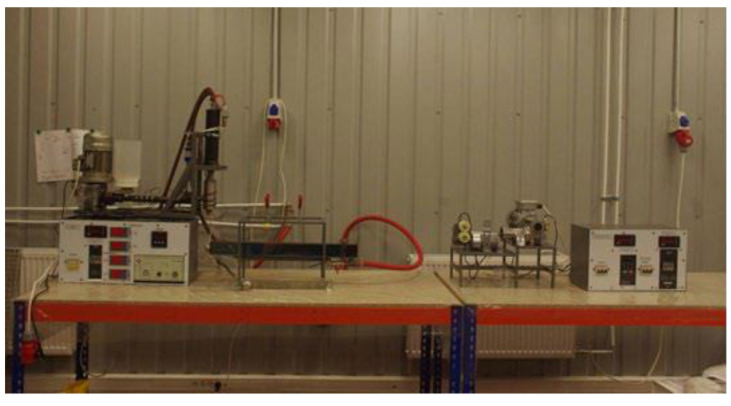
Laboratory extruder with ultrasonic melt treatment.

For obtaining polymer compositions in form of granules was used laboratory extruder with ultrasonic melt (22.4 kHz, the ultrasound power was 300 W) treatment with a screw diameter of 16 mm and a screw length of 400 mm; the screw speed—90 rpm (Figure 2).

The temperature conditions for processing the original polymers are presented in Table 1.

The ultrasonic treatment of PCM melts was used for the more uniform distribution of the antimicrobial additive in the material. In addition, this effect makes it possible to reduce the processing temperature of the compositions in extruder zones 2, 3, and 4 by 10 °C. The effect of reducing the processing temperature of the compositions is associated with a decrease in pressure in the head, apparent viscosity and activation energy of the polymer melt flow under ultrasonic action. These regularities are presented in [29] and confirm the effect of lowering the temperature of polymer processing in the presence of ultrasonic treatment at constant apparent viscosity.

**Figure 3:**
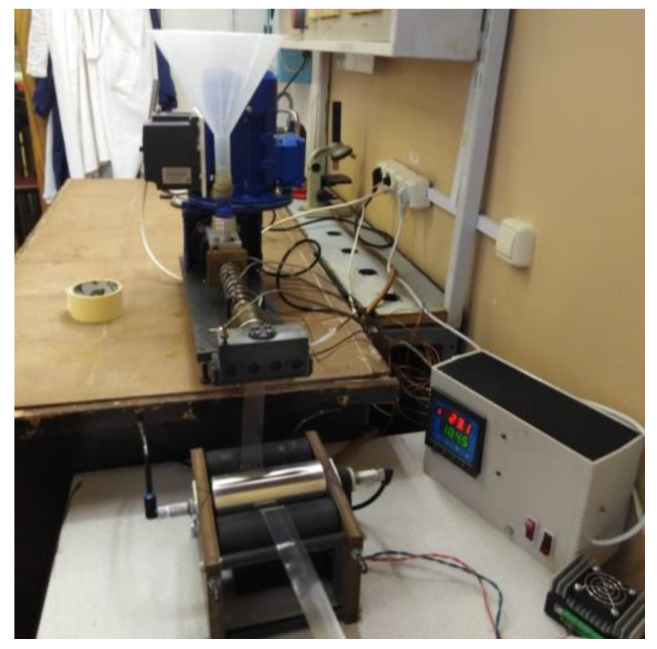
Laboratory extruder for forming flat film.

For obtaining samples of film materials, the extrusion screw of the laboratory extruder with a length of 300 mm was used, the screw diameter (d) was 12 mm; screw speed was 90 rpm (Figure 3).

Temperature conditions for processing PCM granules are presented in Table 2.

### 2.3. Research Methods

#### 2.3.1. Appearance

The appearance of the obtained granules was assessed visually. The granules should be smooth, regular cylindrical in shape. The color of the granules can vary from white to yellow, which is determined by the color of the starting materials. The size of the granules (diameter, length) was determined with a calipers.

Visual assessment of the appearance of the obtained film materials was carried out in order to determine the color of the outer and inner surfaces and the presence of surface defects. The film must be free from cracks, pressed folds, tears and through holes. The color of the film samples must match the color of the original granules. The thickness of flat films was determined according to ISO 4593:1993.

#### 2.3.2. Studying the Structure

The structural and morphological properties of the samples were studied by electron microscopy. The JSM-U3 scanning electron microscope (JEOL, Tokyo Japan) was used for the study. The increase was 70,000 times.

#### 2.3.3. Determination of Physico–Mechanical Properties

Determination of the physico–mechanical properties of polymer samples was carried out in accordance with ISO 527-3:2018. The universal testing machine RM-50 (Moscow, Russia) was used with the fixation of the breaking stress and deformation at rupture. The stretching of the film samples was carried out at the air temperature of 23 ± 2 °C and the relative air humidity of 50% ± 5%. The deformation rate was 100 mm/min. The limit of the permissible value of the load measurement error during the forward stroke did not exceed ± 1% of the measured load. The studies were carried out in five replicates.

#### 2.3.4. Study of Antimicrobial Properties

The antimicrobial properties of polymeric materials were evaluated in relation to microorganisms: *Escherichia coli M 17 (E. coli)*, *Candida albicans ATCC 2091 (C. albicans)*, *Penicillium commune F-4486 (P. commune)* by the disk diffusion method. The experimental technique is described in detail in [23]. Samples of polymeric materials in the form of disks (*d* = 20 mm) were placed in Petri dishes with inoculated solid agar medium. Petri dishes with the studied samples were placed in the TS-1/20 SPU thermostat (Russia) at the temperature of 37 ± 1 °C for 48 h (for *E. coli, C. albicans*) and at the temperature of 28 ± 1 °C for 72 h (for *P. commune*). After 24 h, the intermediate inspection of the plates was performed. The development of microorganisms on the materials’ surface and the presence of the inhibition growth zone were visually assessed. The experiment was held three times.

Additionally, studies of the resistance of materials to the *Aspergillus niger 82 (A. niger)* strain were carried out under conditions of mold spore contamination and the absence of mineral and organic pollutants at high humidity (over 90%) and a temperature of 29 ± 1 °C. Inoculation of the samples was carried out by spraying the suspension of the mold spores on the surface of the film, preventing droplets from merging. The experimental technique is described in detail in [23]. The test duration was 14 days. The surface was evaluated after the incubation time expiration by light microscopy at the increase of 100×. The experiment was carried out three times.

#### 2.3.5. Biodegradability Assessment

To assess the biodegradability of the obtained materials, the composting method was used, which is described in detail in the work [23] and ISO 14855. Samples of polymeric materials were placed in a container with vermicompost (TU 0391-11158096-2002) with the moisture content of at least 50% of its maximum moisture capacity. A layer of vermicompost was poured on top of the samples, loosely closed containers were placed in a chamber at the temperature of 23 ± 2 °C and the humidity of 60% ± 5%. Temperature and humidity were monitored throughout the composting process. The composting time for polymeric materials was 4 months. The degree of PCM biodegradation was determined by the change in the mass of the samples and the physic-mechanical properties during composting. The calculation of the degree of biodegradation of the compositions was carried out according to the formula:

(1) $$\Delta =\frac{a_{1}-a_{0}}{a_{0}}\times 100\%,$$

where *a*_1_ is the measured value before composting and *a*_0_ is the measured value after composting.

The change in the relative elongation upon rupture of PCM samples before and after composting was used as the assessment criterion. The change in the relative elongation at break of the film samples was determined according to ISO 527-3:2018, for this we used the testing machine RM-50 (Moscow, Russia).

#### 2.3.6. Differential Scanning Calorimetry (DSC)

The methods of differential scanning calorimetry was performed on a DSC-500 device (Moscow, Russia). The heating rate of the sample 3 °C/min.

### 2.4. Statistics

The results are presented in the format M ± Sd (M—is the average value of the studied trait, Sd—is the standard deviation). Statistical processing of the results was performed using the IBM SPSS Statistics Program 20 (SPSS Inc., Chicago, IL, USA).

## 3. Results and Discussion

### 3.1. Appearance

The obtained samples of PCM granules had regular cylindrical shape with smooth surface. Pure PBAT granules are white and opaque; granules PBAT + BBE are white-yellow, also opaque. The length of the obtained PCM granules with and without the ultrasonic treatment is 4 ± 2 mm, the diameter is 2 ± 1 mm.

The samples of the obtained film materials do not have cracks, pressed folds, breaks and through holes; they are quite dense, and have a smooth glossy surface. The samples of PBAT-based film materials are opaque, white. The introduction of BBE into the PBAT polymer matrix leads to the change in the color of the obtained materials. At the BBE concentration of 1 wt % of the film became noticeably more yellow. For comparison, polyethylene (PE) granules and films are colorless and transparent [23]. The thickness of the obtained samples of PCM films is presented in Table 3.

### 3.2. Studying the Structure

**Figure 4:**
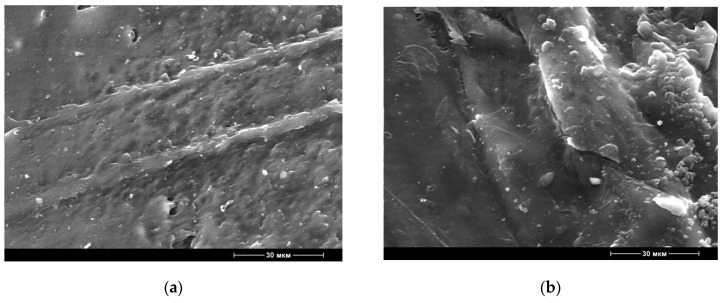

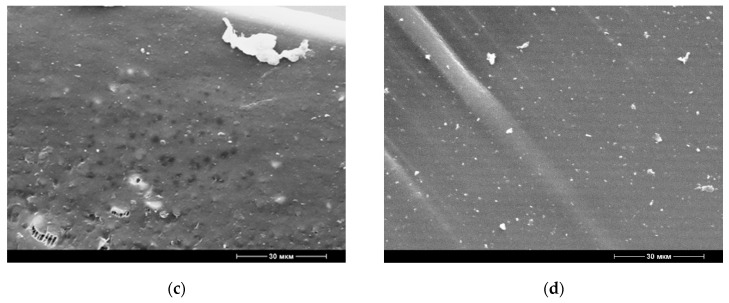
Micrographs of the surface structure of the samples of the materials PBAT (70,000×): (**a**) PBAT, obtained without ultrasonic treatment; (**b**) PBAT+BBE, obtained without ultrasonic treatment; (**c**) PBAT, obtained with ultrasonic treatment; (**d**) PBAT+BBE, obtained with ultrasonic treatment.

The film samples, obtained without the ultrasonic treatment were distinguished by the presence of the agglomerated filler. Due to the ultrasonic treatment effect on the composition melts, the antimicrobial additive in the samples of the obtained films is well dispersed in the polymer matrix. This is confirmed by the electron microscopy images (Figure 4).

The micrographs clearly show the absence of filler agglomerates. Similar results were obtained in the study of composites based on PE in [23].

### 3.3. Determination of Physic-Mechanical Properties

Experimental data of studies of PCM samples in terms of physico–mechanical characteristics are presented in Table 4.

Based on the data obtained, it can be concluded that PBAT-based film materials have higher tensile strength, compared to traditional PE [23]. And the introduction of BBE into the PBAT matrix leads to the slight decrease in the values of the physico–mechanical characteristics of PCM. The ultrasonic treatment of composition melts leads to the decrease in the strength of the samples under study, but at the same time to the increase in the elongation at break. This is likely due to the more even distribution of the antimicrobial additive during the ultrasonic treatment.

To confirm the amorphization process, studies were carried out with the methods of differential scanning calorimetry. It has been found out, that the melting heat is less by 30% for the samples of polymer compositions, obtained with ultrasonic treatment, than for samples obtained without ultrasonic treatment (Table 5).

### 3.4. Study of Antimicrobial Properties

The results of evaluating the antimicrobial properties of the studied samples of materials in relation to *E. coli*, *C. albicans*, and *P. commune* are presented in Table 6.

Based on the data obtained, it was found out, that the introduction of BBE into the PBAT matrix affects the antimicrobial properties of this composition. At the same time, the ultrasonic treatment has no effect on the antimicrobial activity of the test samples in relation to the selected cultures of microorganisms.

The antimicrobial properties of PBAT + BBE materials were observed for all cultures of microorganisms: *E. coli*, *C. albicans*, and *P. commune* in comparison with control samples. At BBE concentration of 1.0 wt % during the first 48 h, no culture growth was observed on the surface of the materials, i.e., PBAT materials containing 1 mass. % BBE, have bacteriostatic and fungistatic properties. Similar results were obtained in the study of compositions of PE with BBE in [23]. Thus, it can be concluded that modification with birch bark extract gives antimicrobial properties to polymers of various chemical nature.

**Figure 5:**
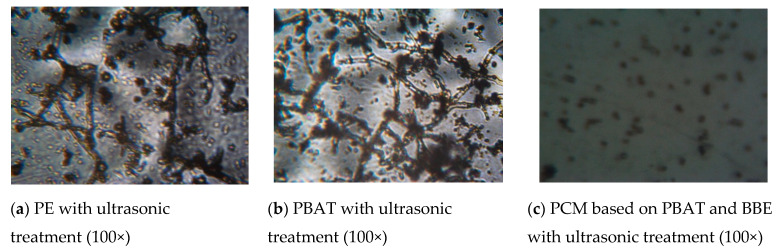
Micrographs of the surface of inoculated materials on the 14th day of exposure to *Aspergillus niger*.

Additionally, the stability of PBAT + BBE materials obtained with the ultrasonic treatment of melts and exposure to high humidity (more than 90%) at 29 ± 1 °C was assessed against *A. niger* according to the degree of fungi development on the film surface under a microscope (Figure 5).

Earlier in the work [23], it was revealed that on the surface of materials based on PE without BBE, after 14 days of exposure under conditions of forced superficial inoculation with *A. niger* at the temperature of 29 ± 1 °C and the air humidity of more than 90%, mycelium is formed (Figure 5a).

In the study, the following results were obtained. Mycelium development was observed on the surface of PBAT-based materials after 14 days of exposure to *A. niger* (Figure 5b); on the surface of PBAT + BBE materials, complete destruction of mycelium was observed (Figure 5c). This indicates the resistance of materials with BBE against the strain of *A. niger*. Similar data were obtained in the work [23].

### 3.5. Biodegradability Assessment

**Figure 6:**
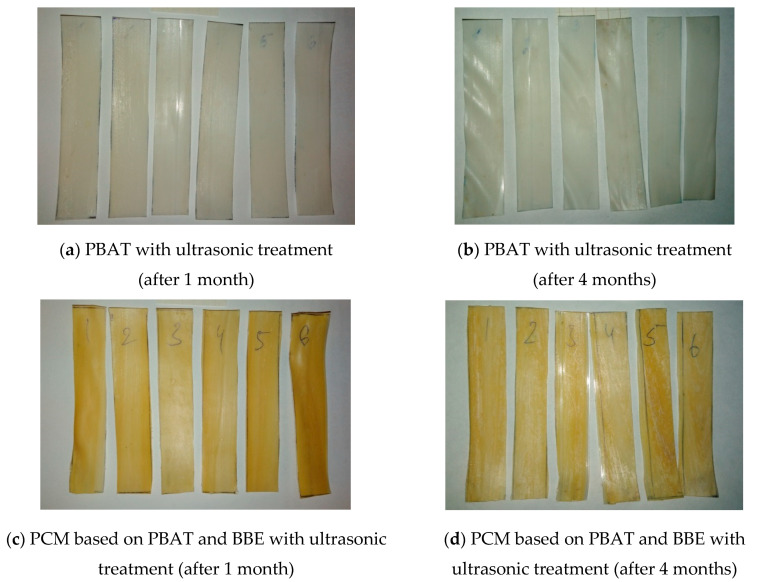
Changes in the appearance of the obtained PCM samples during the composting period.

To assess the biodegradability of the obtained PCMs, a composting study of PCMs was carried out. The study was carried out for 4 months; the change in the appearance of the obtained film materials is shown in Figure 6.

The weight of samples of polymer compositions with BBE decreased by 10.6% during 4 months of composting, and for polymer compositions without BBE it was 21.2%.

The change in elongation at break after 4 months of composting was used as the criterion for assessing biodegradability. The results are presented in Table 7. After the specified period, the film samples look more fragile with the warped surface, PCM with the antimicrobial additive discolored slightly.

In contrast to PE [33], with the change in elongation at break after 4 months of composting is about 3–5%, in this work all PCM samples based on PBAT showed the change in this criterion for assessing biodegradability by more than 50%. This confirms the data that materials based on the studied biopolymer are biodegradable. It should be noted that the PCM samples, based on pure PBAT, showed the more noticeable change in elongation at break after composting, than PBAT samples with the antimicrobial additive. This indicates that the introduction of BBE into the biopolymer matrix slows down the decomposition of the studied materials in the environment, probably due to the fact that the antimicrobial additive prevents the development of microorganisms on the PCM surface and the biodegradation process proceeds due to the hydrolysis of the PBAT polymer matrix.

At the same time, it should be noted that the ultrasonic treatment of the melts of polymer compositions affects the biodegradability of the materials under study. PCM samples obtained with the ultrasonic treatment are destroyed faster than samples obtained without the ultrasonic treatment. It was suggested that the ultrasonic treatment of the melt leads to “amorphization” of the structure of the polymer matrix, which accelerates the biodegradation process. Using the method of accelerated biodegradation, developed and registered at FSBEI VO “MGUPP” (know-how of MGUPP), it was found that the materials under study, obtained with the ultrasonic treatment of the melts, based on pure PBAT, will decompose within 1 year, and materials based on PBAT and BBE—within 2–3 years.

## 4. Conclusions

The effect of the addition of BBE on the antimicrobial properties of PBAT and its biodegradability was investigated. Samples of film materials based on polymer composites were obtained by extrusion with and without the ultrasonic treatment of the melts. Introduction of PBAT BBE 1 mass. % into the polymer matrix does not affect its physico–mechanical properties significantly. Using electron microscopy, it was found out that the ultrasonic treatment of melts of polymer compositions contributes to the production of materials with the uniform distribution of composition components. It was found out, that the introduction of 1 mass. % BBE imparts antimicrobial properties to the studied biopolymer. In this case, the composting period of the modified biopolymer is increased compared to pure PBAT.

Thus, we can conclude that the developed material can potentially be used as a packaging material for food products. The use of PBAT with BBE, which combines antimicrobial properties and biodegradability, will allow preserving the quality of the food product for the longer time and reducing the burden on the environment. However, additional research is required, first of all, the study of the sanitary-hygienic and rheological characteristics of this composition.

## Figures and Tables

**Table 1:** Temperature conditions for processing polymer composite materials (PCM) granules in the extruder (*d* = 16 mm).

Temperature in the Extruder by Zone, °C				Polymer Compositions
Zone 1	Zone 2	Zone 3	Zone 4 *	
120	170	180	200	PBAT with US
120	160	180	190	PCM based on PBAT and BBE with US
120	170	190	210	PBAT without US
120	160	190	200	PCM based on PBAT and BBE without US

* Zones 1–3 represent zones in the extruder; zone 4 is an extrusion head.

**Table 2:** Temperature conditions for processing PCM in the extruder (*d* = 12 mm).

Temperature in the Extruder by Zone, °C				Polymer Compositions
Zone 1	Zone 2	Zone 3	Zone 4 *	
120	170	180	200	PBAT with US
120	160	180	190	PCM based on PBAT and BBE with US
120	170	190	210	PBAT without US
120	160	190	200	PCM based on PBAT and BBE without US

* Zones 1–3 represent zones in the extruder; zone 4 is an extrusion head.

**Table 3:** Thickness of the obtained PCM film samples.

Polymer Compositions	Thickness, Microns
PBAT with US	31.73 ± 1.63
PCM based on PBAT and birch bark extract (BBE) with US	35.61 ± 1.92
PBAT without US	30.52 ± 2.68
PCM based on PBAT and BBE without US	33.85 ± 1.89

**Table 4:** Physico–mechanical properties of polymer compositions.

Polymer Compositions	Breaking Stress, MPa	Elongation at Break, %
PBAT with US	21.8 ± 3.2	678 ± 148
PCM based on PBAT and BBE with US	17.6 ± 1.9	570 ± 136
PBAT without US	24.2 ± 2.1	520 ± 85
PCM based on PBAT and BBE without US	19.7 ± 2.8	410 ± 69

**Table 5:** The melting heat for polymer compositions.

Polymer Compositions, Obtained with Ultrasonic Treatment or without Ultrasonic Treatment	The Melting Heat for the Polymer Compositions
PBAT BBE with US	71 ± 3
PBAT BBE without US	81 ± 3
PBAT with US	67 ± 3
PBAT without US	78 ± 2

**Table 6:** Results of visual assessment of the surface of polymer compositions, inoculated within 24–48 h.

Polymer Compositions	Visual Assessment		
	*Escherichia coli*	*Candida albicans*	*Penicillium commune*
PBAT with US	growth on surface	growth on surface	growth on surface
PCM based on PBAT and BBE with US	lack of surface growth	lack of surface growth	lack of surface growth
PBAT without US	growth on surface	growth on surface	growth on surface
PCM based on PBAT and BBE without US	lack of surface growth	lack of surface growth	lack of surface growth

**Table 7:** Results of determining the degree of biodegradation of the studied PCM.

Polymer Compositions	Change in Elongation at Break after Composting for 6 Months, %
PBAT with US	62 ± 7
PCM based on PBAT and BBE with US	51 ± 5
PBAT without US	56 ± 7
PCM based on PBAT and BBE without US	45 ± 4
